# Expert-level Automated Biomarker Identification in Optical Coherence Tomography Scans

**DOI:** 10.1038/s41598-019-49740-7

**Published:** 2019-09-19

**Authors:** Thomas Kurmann, Siqing Yu, Pablo Márquez-Neila, Andreas Ebneter, Martin Zinkernagel, Marion R. Munk, Sebastian Wolf, Raphael Sznitman

**Affiliations:** 1 ARTORG Center, University of Bern, Bern, Switzerland; 2 Department of Ophthalmology, Inselspital, University Hospital, University of Bern, Bern, Switzerland

**Keywords:** Diagnostic markers, Translational research, Biomedical engineering

## Abstract

In ophthalmology, retinal biological markers, or *biomarkers*, play a critical role in the management of chronic eye conditions and in the development of new therapeutics. While many imaging technologies used today can visualize these, Optical Coherence Tomography (OCT) is often the tool of choice due to its ability to image retinal structures in three dimensions at micrometer resolution. But with widespread use in clinical routine, and growing prevalence in chronic retinal conditions, the quantity of scans acquired worldwide is surpassing the capacity of retinal specialists to inspect these in meaningful ways. Instead, automated analysis of scans using machine learning algorithms provide a cost effective and reliable alternative to assist ophthalmologists in clinical routine and research. We present a machine learning method capable of consistently identifying a wide range of common retinal biomarkers from OCT scans. Our approach avoids the need for costly segmentation annotations and allows scans to be characterized by biomarker distributions. These can then be used to classify scans based on their underlying pathology in a device-independent way.

## Introduction

**Figure 1:**
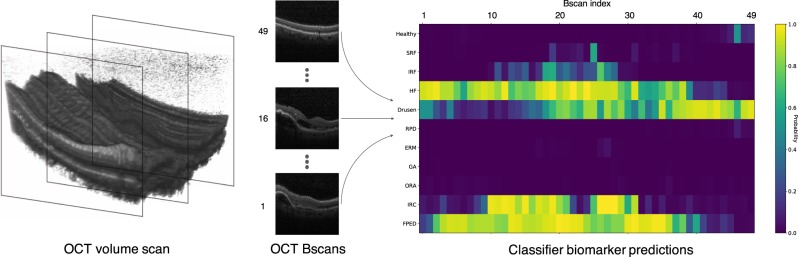
Evaluation of an OCT volume scan (left) using our proposed automated biomarker identification method. Individual cross-sections, or Bscans (middle), are processed and predictions on eleven biomarkers are given per cross-section (right). The color coding indicate the likelihood of a specific biomarker being present in a given cross-section.

Optical Coherence Tomography (OCT) scans play an important role in diagnosing and managing sight-threatening macular diseases such as age related macular degeneration (AMD) and diabetic macular edema (DME). By imaging the retina at micrometer resolution, OCT has given ophthalmologists the ability to visualize retinal structures in three dimensions (see Fig. 1). Yet, detailed analysis of OCT scans in clinical routine is time-consuming even for experienced physicians^1^. With over 30 million OCT scans acquired annually worldwide and an increasing prevalence of chronic eye conditions, the human resources and expertise needed to assess OCT images, today and in years to come, are simply overwhelming^2,3^.

Machine learning provides a pathway to automate inspections of medical imaging such as OCT scans. By using datasets of annotated examples, trained machine learning algorithms are not only faster at assessing scans, but also more cost effective when compared to human counterparts. These advantages have led to a surge of machine learning based methods for retinal image analysis^4–6^. These include techniques that perform automated diagnosis^7^, morphological shape estimation^8–12^, treatment outcome estimation^13–15^ and clinical referral support^16^. Broadly, these developments have hinged on clinical insights, novel machine learning techniques and large amounts of OCT scans.

Biological markers, or *biomarkers* of the retina, have traditionally played a central role in both clinical routine and research^17^. For example, monitoring fluctuations of fluid biomarkers using OCT is an essential part of the standard of care for managing chronic retinal conditions, while other biomarkers have been linked to how well patients respond to treatments^18^. However, given that there are dozens of established biomarkers, their identification is both time consuming and challenging due to their number, size, shape and extent. Furthermore, other morphological markers yet to be identified may have a significant impact on patients’ outcomes, needed treatment and prognosis.

At the core of this work, we hypothesize that an automated method can identify biomarkers reliably and can also help answer routine clinical questions. To show this, we present a machine learning method that automatically identifies a wide range biomarkers in OCT scans. Our approach learns to identify biomarkers without needing to be shown where these are located in training scans, and obviates the need for burdensome segmentation annotations. By training our algorithm this way, our method is not only capable of identifying biomarkers more consistently then experienced experts, it also allows a robust representation of retinal characteristics that can be used to identify pathologies in OCT scans acquired with different OCT devices.

## Methods

### Data and biomarkers

To train our method, we acquired 6 × 6 mm volume OCT scans using the same OCT device type (Spectralis, Heidelberg Engineering, Germany). The scan protocol included 49 Bscan for each volume scan and an ART setting of 9 (each Bscan was averaged 9 times). Pathologies included diabetic retinopathy with and without DME, and early, intermediate and late AMD. In total, 327 patients from the outpatient department of the university eye clinic were included in the study. From these a total of 470 volume scans were collected, resulting in 23’030 OCT Bscans (see Supplementary materials for the patients per disease distribution). As a validation set, we collected an additional cohort of 21 AMD patients using the same device type and scanning protocol. This study was approved by the ethics committee of the Kanton of Bern, Switzerland (KEK-Nr. 093/13), and was conducted in compliance with the tenets of the Declaration of Helsinki. Given the retrospective design of this study, the ethics committee waived the requirement for individual informed consent.

The following morphological biomarkers were considered in this study: subretinal fluid (SRF), intraretinal fluid (IRF), intraretinal cysts (IRC), hyperreflective foci (HF), drusen, reticular pseudodrusen (RPD), epiretinal membrane (ERM), geographic atrophy (GA), outer retinal atrophy (ORA) and fibrovascular pigment epithelial detachment (FPED). A healthy biomarker was also included to denote the lack of any previously mentioned biomarker. Note that these parameters are established predictive OCT biomarkers^18–20^, used to stage diseases and assess disease progression (see Supplementary materials for a detailed definition of each biomarker used in this work).

### Annotation protocol

To annotate which biomarkers were present in individual OCT Bscans, we developed a custom web-based annotation tool to allow fast and convenient annotations (grading) of the image data (see Supplementary video for an illustration of the tool in use). This tool allows OCT volumetric scans to be uploaded and viewed directly in a web browser, with multiple annotators able to grade simultaneously with the same protocol.

A pool of eight experienced annotators from the Bern University Hospital and the Bern Photographic Reading Center contributed to this study. Prior to the annotation collection, all annotators were trained for respective biomarkers on additional scans containing the biomarkers defined. For each Bscan, annotators were instructed to mark which biomarkers were present in the given scan using dedicated buttons (biomarker is present or not) and could also view adjacent Bscans at the same time. A “healthy” grade was assigned when a Bscan contained no other pathological biomarker, while a “can’t grade” grade was given to low quality scans that did not allow confident assessments. For additional consistency, annotators were provided with an annotation manual providing definitions and example images of each biomarker.

Each of the 23’030 Bscans in the training set was annotated by a single annotator randomly selected from the annotator pool. For the validation set, we randomly sampled 49 Bscans for each of the 21 imaged patients. To obtain a broader spectrum of disease stages in this validation set, Bscans were selected from OCT volumes acquired over several years and from both eyes. This resulted in a total of 1029 Bscans, each of which was then graded by at least five graders.

### Bscan-based biomarker classification

We propose to use a convolutional neural network (CNN) classifier to identify which biomarkers are present in any given Bscan. Our method uses a set of OCT Bscans and the associated biomarkers present in that scan to train the method. This is in contrast to recent trends^8,11,12,16^, that use pixel wise segmentations and volumetric information to provide segmentations. Therefor our approach has two advantages: firstly, our method requires annotations that are far simpler to gather, especially with the tool proposed. Secondly, our method assesses individual Bscans, allowing it be used on volumes by repeatedly evaluating scans in volume or be used on individual scans as is often the case in clinics, where each individual scan of the cube/volume scan is assessed for the presence of respective biomarkers such as IRF or SRF.

Our chosen architecture is based on residual convolutional neural networks^21^ with dilated convolutions^22^ and is fully described in the Supplementary material. This choice is motivated by the need to identify a wide range of biomarkers that vary in size, position and extent. For instance, HF biomarkers can occupy a few pixels in size, while the entire Bscan needs to be assesed to assign a healthy label. The use of dilated convolutions is motivated by the fact that many networks such as the ResNet^21^, decrease the image size in the network to increase the receptive field by means of pooling. While maximum pooling may retain the activation of a small biomarkers such as HF, we argue that retaining a large spatial size of the image is advantageous for small to medium sized biomarkers. A drawback of the large spatial size of the image is the reduced receptive field compared to pooling networks. To overcome this, Yu *et al*.^23^ proposed to use dilated convolutions that increase the receptive field more strongly than conventional convolutions using the same number of model parameters. To further mitigate the problem of identifying small biomarkers, we not only use global average pooling after our last convolutional layer, but both global maximum pooling and global average pooling on the same feature maps after concatenating the vectors.

As in previous methods^16,24^, we construct an ensemble of CNNs using cross-validated models and combine the prediction of all models using the unweighted mean of the final output before sigmoid activation (see Supplementary material for more details). We perform 10-fold cross-validation on the training set, where every model is trained on 90% of the training data and validated on 10% of the training data. To train our network, we use the weighted binary cross entropy loss function and Stochastic Gradient Descent. The inputs are 512 × 512 pixel RGB grayscale Bscans. We pre-train the network using ImageNet^25^ and then perform transfer learning on the B-scan task to maximize the classifiers performance.

### Expert and method evaluation

Using the validation set to evaluate how well expert graders perform the task of Bscan grading, we first apply a majority voting scheme using all graders to determine a gold-standard annotation for all biomarkers on all Bscans. At least half of the graders must have been in agreement to imply that a biomarker is in fact present.

To assess the performance of graders, we apply two validation schemes: (1) inter-grader performance and (2) performance compared to the majority vote. Inter-grader performance is assessed by computing the Cohen’s Kappa values of two graders on commonly annotated slices for each biomarker. Performance to the majority vote is evaluated by including all graders to determine the majority vote and then computing the Cohen’s Kappa of a grader to the majority vote. Given that the latter may result in biased results (due to the grader contributing to the majority vote), we also compare performances with each grader when discarded from the majority vote computation.

We then evaluate our method’s capacity to identify biomarkers in Bscans on the same validation set using a variety of performance metrics: Cohen’s Kappa, sensitivity, specificity, average precision and F1 scores. Additional performance information regarding the architecture design choice is also given in the Supplementary materials.

To estimate the generalization performance of our method for the purpose of pathology classification, we make use of the publicly available Duke AMD dataset^26^. This dataset includes OCT volumes from 269 AMD and 115 healthy eyes, acquired using a Bioptigen, Inc SD-OCT imaging system, and AMD or healthy annotations are give on a per volume basis. The imaging protocol of this dataset differs from that of our training set, as every volume contains 100 Bscans. Given the different spatial resolution of 1000 AScans in ±6.7 mm compared to our 512 AScans in ±6 mm, we spatially normalize Bscans from this dataset to 512 × 570 pixels. While our biomarker classifier does not directly predict if a volume has AMD, our method can yield a distribution of biomarkers from a volume when aggregating the predicted biomarkers on each Bscan of a volume. In order to create a classifier out of this distribution, the sum of the sigmoid activated logits is computed for all AMD biomarkers (SRF, IRF, Drusen, RPD, IRC, FPED) and over all Bscans. Note that we do not train on any of the data from this dataset even though it is acquired with a different device than that of the data our method is trained on.

Similarly, we also examine the performance of our biomarker predictor on the public test dataset provided by Kermany *et al*.^27^ for the task of pathology classification. This dataset contains 1000 Bscans from eyes with healthy, CNV, drusen and DME conditions (250 from every category). Data was collected using the Heidelberg Spectralis OCT device. We evaluate the performance of our method on each Bscan and map the set of predicted biomarkers to a pathology category. We do so by using a simple comparison tree: CNV if fluid (SRF, IRF or IRC) is present with either drusen, FPEDs or RPD being present; DME if fluid (SRF, IRF or IRC) is present and neither drusen or PED are present; Drusen if fluid is not present but drusen, FPED or RPD are; healthy otherwise.

## Results

### Expert-level grading of biomarkers

The validation set contained 1029 Bscans of which 1002 Bscans had at least one biomarker annotated in agreement. Bscans with no biomarker agreement were removed from further analysis. On average, grading time of all biomarkers on a single Bscan lasted 24.2 seconds and every Bscan had on average 1.8 ± 0.983 majority voted biomarkers (see Supplementary materials for the complete majority voted statistics).

**Figure 2:**
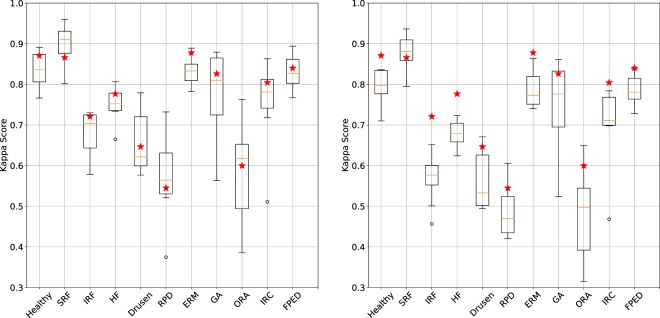
Biomarker grading performance by annotators and our method. For each biomarker, the distribution of human grader Kappa values is illustrated with a dedicated box plot (median, first and third quantiles). The red stars depict the performance of our method. (left) depicts values when the majority includes all graders and (right) when individual graders are discarded from the majority vote.

On average, the mean Kappa value for all biomarkers across graders was of 0.742 ± 0.036, with maximum and minimum scores of 0.781 and 0.674, respectively. When leaving out respective graders to compute majority votes, mean Kappa value across biomarkers decreased to 0.677 ± 0.035, with maximum and minimum scores of 0.710 and 0.607, respectively. Table 1 summarizes the performance of each grader on the validation set, while Fig. 2 illustrates for each biomarker the distribution of Kappa scores across graders. The highest average inter-grader Kappa values were found to be for SRF ($\kappa =0.8367$) and ERM ($\kappa =0.7386$), while the lowest were for Outer Retinal Atrophy ($\kappa =0.365$) and Reticular Pseudodrusen ($\kappa =0.361$).

**Table 1:** Grader assessment on the validation set.

Grader	Sensitivity (leave-out)	Specificity (leave-out)	F1 Score (leave-out)	Mean Kappa	Mean Kappa (leave-out)	# of annotations
1	0.787	0.937	0.763	0.781	0.710	954
2	0.766	0.907	0.710	0.705	0.645	999
3	0.754	0.954	0.736	0.748	0.686	1000
4	0.737	0.949	0.751	0.763	0.701	985
5	0.769	0.934	0.710	0.718	0.653	858
6	0.739	0.952	0.753	0.776	0.703	896
7	0.705	0.936	0.670	0.674	0.607	924
8	0.755	0.952	0.756	0.771	0.709	568
Mean (std)	0.752 (±0.023)	0.941 (±0.015)	0.732 (±0.030)	0.742 (±0.036)	0.677 (±0.035)	Total: 1002

Average sensitivity, specificity, F1-score and mean Kappa values are presented for each grader. Scores when leaving graders out of the major vote (leave-out) are also given.

**Figure 3:**
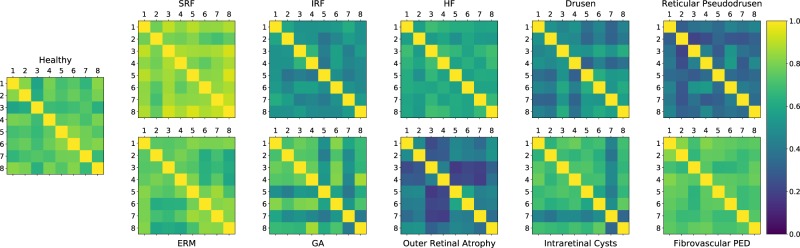
Inter-grader Kappa values for each each biomarker. The 8 grader IDs are along the vertical and horizontal axes, while light and dark colors show high and low Kappa values between any pair of graders, respectively.

Figure 3 illustrates the Kappa value relations between each pair of graders for each biomaker, where lighter colors represent higher Kappa values and darker colors correspond to low values.

### Network-based biomarker grading

Our method yields an average Kappa value of 0.761 ± 0.111 over all biomarkers. The exact exact match ratio (proportion of Bscans where the method predicted the majority vote) was of 0.502 and an at-most-one-wrong ratio (proportion of Bscans where the method predicted the majority vote with at most one biomarker mistake) was of 0.824. Our method achieves the highest Kappa values for ERM ($\kappa =0.877$) and the lowest for Reticular pseudodrusen ($\kappa =0.545$). Figure 2 (left) shows the performance of our method in comparison to human graders for each biomarker and a complete summary of all Kappa scores, as well as sensitivity, specificity, F1 score and average precision are outlined in Table 2. For eight biomarkers, our method has higher Kappa scores than the median human grader. If we also consider our method to be an additional grader and include its predictions to compute the majority vote for each biomarker, then the classifiers mean Kappa increases to 0.817. Figure 2 illustrates how these compare to human graders for each of the biomarkers.

**Table 2:** Performance of the proposed method on the validation set.

Biomarker	# Annotations	Max Kappa	Average Precision	F1 Score*	Sensitivity*	Specificity*
Healthy	165	0.871	0.951	0.965	0.911	0.975
SRF	65	0.866	0.929	0.985	0.962	0.986
IRF	48	0.721	0.810	0.974	0.720	0.987
HF	178	0.776	0.886	0.936	0.839	0.955
Drusen	376	0.647	0.847	0.828	0.729	0.906
Reticular Pseudodrusen	153	0.545	0.669	0.882	0.614	0.931
ERM	140	0.877	0.959	0.971	0.911	0.980
GA	62	0.826	0.882	0.979	0.806	0.991
Outer Retinal Atrophy	200	0.599	0.732	0.863	0.633	0.934
Intraretinal Cysts	54	0.804	0.883	0.980	0.815	0.989
Fibrovascular PED	359	0.840	0.963	0.925	0.866	0.963
All	1002	0.761	0.881	0.808	0.801	0.964

* indicates that the classifier threshold taken was at the maximum Kappa value.

**Figure 4:**
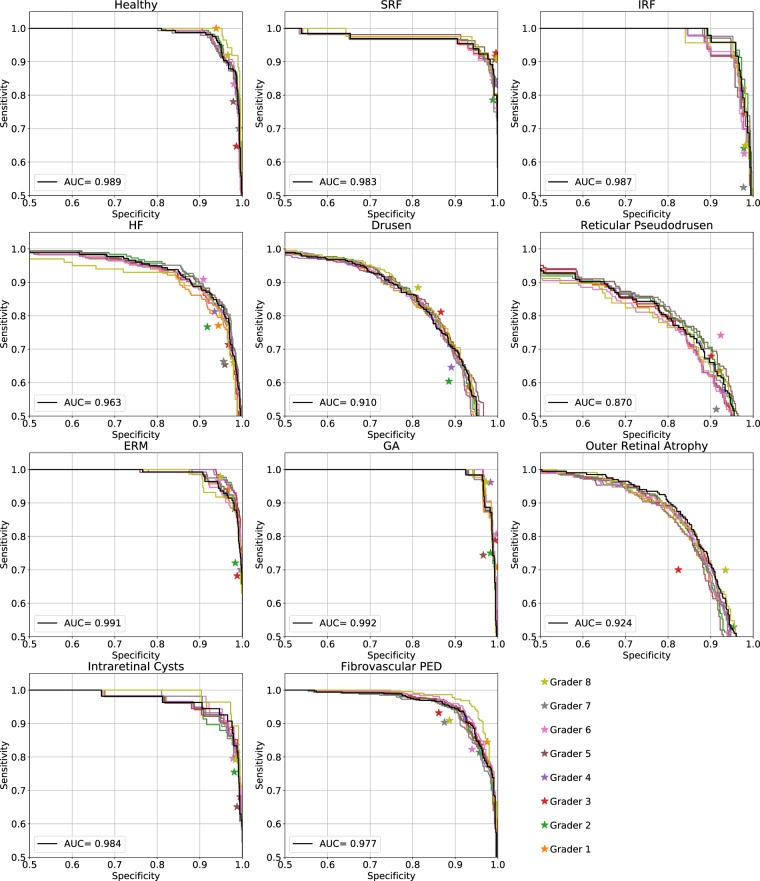
Receiver operating characteristic curves for all biomarkers. Stars denote the leave grader out performance. The black ROC curve compares our method with the majority voted ground truth of all graders. Colored curves denote the comparison of the automatic classifier with the majority vote of corresponding leave-grader out evaluation.

**Figure 5:**
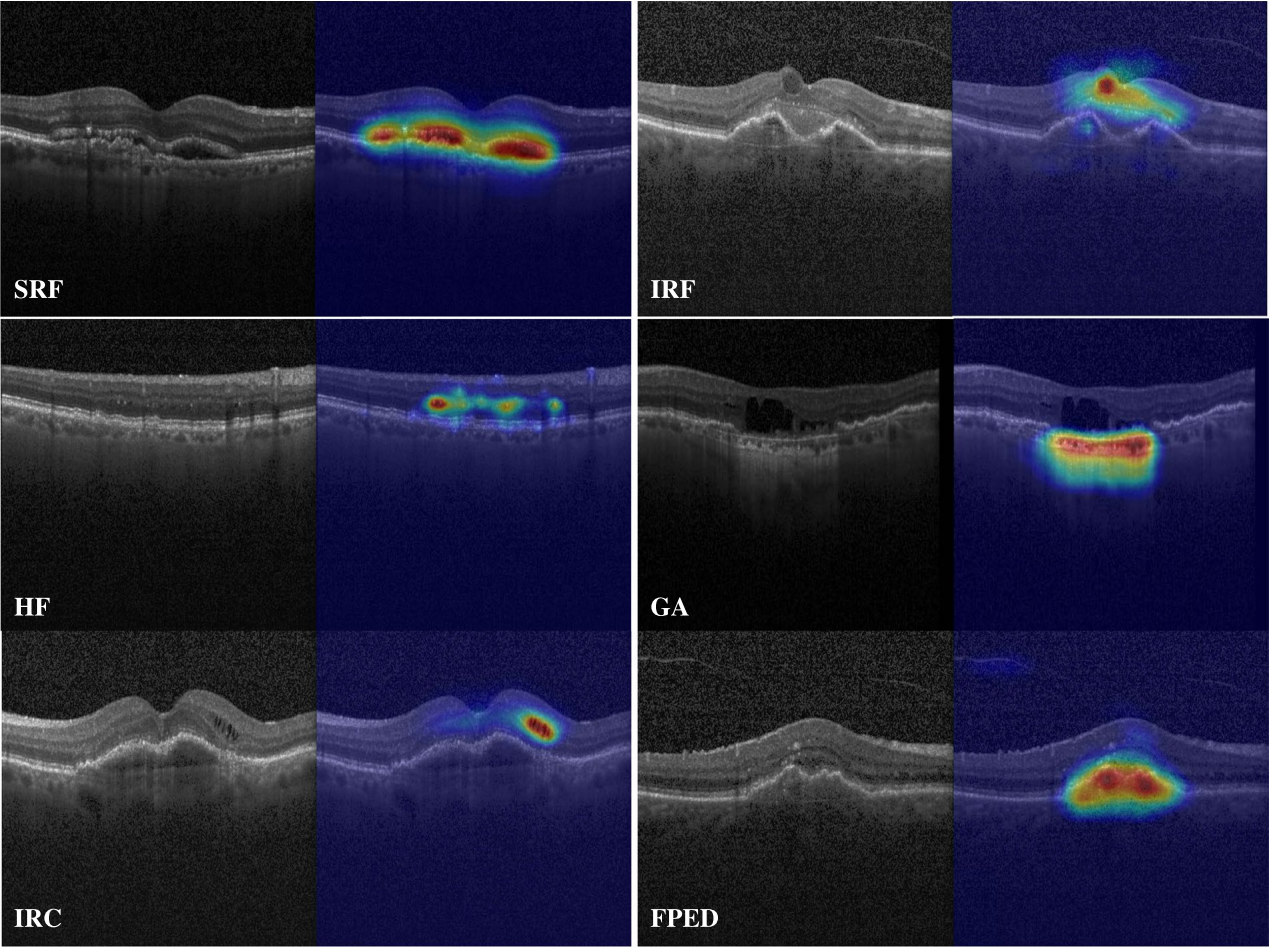
Classifier activation maps for different biomarkers on selected examples. Red regions illustrates regions of the image that are automatically found by our method to correctly predict the corresponding biomarker.

Figure 4 shows the Receiver operating characteristic (ROC) curves of all biomarkers. Here, we compute eight different leave-grader-out majority votes and compare them with our method’s prediction, whilst plotting the graders’ specificity and sensitivity. Using class activation maps^28^, we illustrate in Fig. 5 what part of the image data contributes to the classifier’s decisions on selected examples. For each example, we highlight in warm colors (red) regions of high relevance in the classifier decision.

### Automated pathology classification

**Figure 6:**
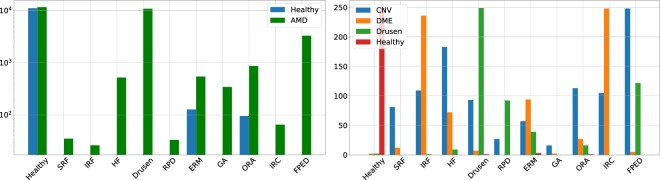
Distribution of predicted biomarkers for the Duke Dataset (left) and Kermany Dataset (right). Using a threshold on the biomarker probability (*p* ≥ 0.5) to indicate presence, values are aggregated with occurrence over all scans and within each volume.

Using automatically identified biomarkers to classify Bscans based on pathology, Fig. 6 outlines the distribution of biomarker distributions for both the Duke (left) and Kermany (right) datasets. In both cases, the distributions are split based on the provided pathological groupings.

In the Duke dataset case, for all biomarkers except “Healthy”, significant differences in quantities of biomarkers were found. Using our approach to classify these images as AMD or healthy, we report an AUC of 0.9981 using all AMD biomarkers and 0.9992 using only the drusen biomarker, compared to reported state-of-the-art methods performance of 0.999^29^ and 0.997^30^ on this same dataset. At a false positive rate of 0% and 1.7%, our approach achieves a true positive rate of 96.2% and 99.3%, respectively. We also achieve a maximum accuracy of 96.7% on the Kermany data set, while Kermany *et al*.^27^ reported an accuracy of 96.6%. When classifying healthy from pathological Bscans, we report an accuracy of 99.8% compared to 99.1% previously reported^27^.

## Discussion

We have presented a machine learning method capable of identifying a wide range of biomarkers from OCT Bscans of the retina. Our method is based on convolutional neural networks and is trained using a heterogeneous cohort of treated patients with chronic conditions. We show that our method is not only capable of identifying referenced biomarkers with high reliability, but that these can then be used to classify scans into pathological categories at state-of-the-art levels.

One of the unique qualities of our approach is that it is trained using annotations that can be collected in a matter of seconds per Bscan. That is, the annotations used are simple, only carrying information as to biomarker presence. In contrast to recent methods that have looked to fully locate and delineate relevant anatomical and pathological biomarkers^16^, our approach is significantly less time consuming in terms of data preparation. In addition, segmentation annotations may be error prone^31^, as these are usually performed by a single grader and may not serve as an appropriate gold-standard for accurate training of a classifier^32^. Consequently, the annotations needed by our method can be easily gathered with an appropriate interface, such as the one we propose, and allows for much larger number of different scans to be annotated in the same amount of time.

We have also demonstrated here that the task of biomarker identification from Bscans is in itself a challenging one even for experts. The use of experienced graders from a Reading Center showed that depending on the biomarker, a significant variance in grading outcomes can be expected^33^. While this variance could be linked to a number of factors such as adherence to the biomarker definition, size, extent of the biomarker in the image or image quality, the overall inter-grader reliability of most biomarkers remains quite high. At the same time, our approach provides grading performances that rivals, if not surpasses, experienced graders on virtually all biomarkers evaluated here. This opens the possibility for automated systems for clinical decision support in routine clinical care or validation of large clinical studies.

Our results also shows that characterizing OCT Bscans with clinically known morphological descriptors can provide valuable information for OCT scan classification, supporting the result of De Fauw *et al*.^16^. Whether on single Bscans, or volumetric scans, our approach attains state-of-the-art performances on classifications tasks, by leveraging biomarkers found with our method. The fact that our approach operates on a Bscan level gives way for analysis on even larger cohorts of data as many clinical sites only acquire Bscans for the moment.

Surprisingly, our state-of-the-art performance of pathology classification on the two public datasets evaluated involved OCT scans from multiple devices. In the past, the ability to use machine learning methods on different machines had already been shown in the context of medical scans^34^ and OCT images^16^. What sets our result apart from those however, is that our method is only trained with images from a single device, and yet provides a rich enough biomarker characterization (see Fig. 6) to classify scans acquired with other devices and imaging protocols. This suggests that our histogram representation is to a certain extent robust enough to changes in image variability across imaging devices.

Naturally, a limitation of our method and its use of presence information for training is that it does not provide an intuitive way to quantify biomarkers (in millilitres or in occurrences). This capability could be essential to assess improvement and worsening of underlying pathology using specific biomarkers (IRF and SRF). However, in daily clinical practice, physicians often aim for disease stability and usually treatment decisions are based on presence or absence of disease activity, and disease activity markers, irrespective of whether there is more or less fluid compared to the patient’s last visit^35–37^.

Interestingly however, biomarker activation maps such as those illustrated in Fig. 5 provide both a visual representation of the reasoning of the classifier and a coarse delineation of biomarkers. This latter could potentially serve as a proxy for quantification of biomarkers. In addition, by evaluating each Bscan with our approach, a coarse quantification can still be determined, whereby our approach still allows for repeated detection of a specific biomarker on multiple Bscans to be quantified.

A further limitation of this work includes the restricted validation set used. While we considered 1029 Bscan from 21 patients to be sufficiently large in this work, a similar analysis with a larger cohort of patients would further validate the approach. In particular, evaluations on patients with a variety of diseases and treatment would show evidence of greater generalization to a wider range of clinically relevant cases. Last, our approach does not leverage the fact Bscans are highly correlated to adjacent BScan in a volume. An improved classifier would most likely result if it considered inter-Bscan relations. We plan to explore solutions to these limitations in the future.

## Data Availability

The datasets generated during and/or analyzed during the current study are not publicly available due to privacy constraints. The data may however be available from the University Hospital Bern subject to local and national ethical approvals.

## Supplementary information

Supplementary Materials

supplementary video

## References

[1] Foot B, MacEwen C (2017). Surveillance of sight loss due to delay in ophthalmic treatment or review: frequency, cause and outcome. Eye.

[2] Bourne R (2017). Magnitude, temporal trends, and projections of the global prevalence of blindness and distance and near vision impairment: a systematic review and meta-analysis. Lancet Global Health.

[3] Schmidt-Erfurth U, Klimscha S, Waldstein SM, Bogunovic H (2017). A view of the current and future role of optical coherence tomography in the management of age-related macular degeneration. Eye.

[4] Poplin R (2018). Prediction of cardiovascular risk factors from retinal fundus photographs via deep learning. Nature Biomedical Engineering.

[5] Gulshan V (2016). Development and validation of a deep learning algorithm for detection of diabetic retinopathy in retinal fundus photographs. JAMA.

[6] Venhuizen FG (2018). Deep learning approach for the detection and quantification of intraretinal cystoid fluid in multivendor optical coherence tomography. Biomed. Opt. Express.

[7] Lee CS, Baughman DM, Lee AY (2017). Deep learning is effective for classifying normal versus Age-Related macular degeneration OCT images. Ophthalmology Retina.

[8] Apostolopoulos, S., De Zanet, S., Ciller, C., Wolf, S. & Sznitman, R. Pathological OCT retinal layer segmentation using branch residual U-Shape networks. In *Medical Image Computing and Computer-Assisted Intervention*, *MICCAI 2017*, 294–301 (Springer International Publishing, 2017).

[9] Montuoro A, Waldstein SM, Gerendas BS, Schmidt-Erfurth U, Bogunović H (2017). Joint retinal layer and fluid segmentation in OCT scans of eyes with severe macular edema using unsupervised representation and auto-context. Biomed. Opt. Express.

[10] Fang L (2017). Automatic segmentation of nine retinal layer boundaries in OCT images of non-exudative AMD patients using deep learning and graph search. Biomed. Opt. Express.

[11] Lee CS (2017). Deep-learning based, automated segmentation of macular edema in optical coherence tomography. Biomed. Opt. Express.

[12] Ji Z, Chen Q, Niu S, Leng T, Rubin DL (2018). Beyond retinal layers: A deep voting model for automated geographic atrophy segmentation in SD-OCT images. Transl. Vis. Sci. Technol..

[13] Vogl W-D (2017). Analyzing and predicting visual acuity outcomes of Anti-VEGF therapy by a longitudinal mixed effects model of imaging and clinical data. Invest. Ophthalmol. Vis. Sci..

[14] Vogl, W.-D. *et al*. Spatio-Temporal signatures to predict retinal disease recurrence. In *Information Processing in Medical Imaging*, 152–163 (Springer International Publishing, 2015). 10.1007/978-3-319-19992-4_12 26221672

[15] Bogunovic H (2017). Prediction of Anti-VEGF treatment requirements in neovascular AMD using a machine learning approach. Invest. Ophthalmol. Vis. Sci..

[16] De Fauw J (2018). Clinically applicable deep learning for diagnosis and referral in retinal disease. Nat. Med..

[17] Sun JK (2014). Disorganization of the retinal inner layers as a predictor of visual acuity in eyes with center-involved diabetic macular edema. JAMA Ophthalmol..

[18] Zur D (2018). OCT biomarkers as functional outcome predictors in diabetic macular edema treated with dexamethasone implant. Ophthalmology.

[19] Phadikar P (2017). The potential of spectral domain optical coherence tomography imaging based retinal biomarkers. Int J Retina Vitreous.

[20] Gerendas BS (2018). Predictive imaging biomarkers relevant for functional and anatomical outcomes during ranibizumab therapy of diabetic macular oedema. Br. J. Ophthalmol..

[21] He, K., Zhang, X., Ren, S. & Sun, J. Deep residual learning for image recognition. *ArXiv* 1512.03385 (2015).

[22] Yu, F. & Koltun, V. Multi-Scale context aggregation by dilated convolutions. *ArXiv* 1511.07122 (2015).

[23] Yu, F., Koltun, V. & Funkhouser, T. Dilated residual networks. *ArXiv* 1705.09914 (2017).

[24] Titano JJ (2018). Automated deep-neural-network surveillance of cranial images for acute neurologic events. Nat. Med..

[25] Russakovsky, O. *et al*. ImageNet large scale visual recognition challenge. *ArXiv* 1409.0575 (2014).

[26] Farsiu S (2014). Quantitative classification of eyes with and without intermediate age-related macular degeneration using optical coherence tomography. Ophthalmology.

[27] Kermany DS (2018). Identifying medical diagnoses and treatable diseases by Image-Based deep learning. Cell.

[28] Selvaraju, R. R. *et al*. Grad-CAM: Visual explanations from deep networks via Gradient-Based localization. In *ICCV*, 618–626 (2017).

[29] Rasti R, Rabbani H, Mehridehnavi A, Hajizadeh F (2018). Macular OCT classification using a Multi-Scale convolutional neural network ensemble. IEEE Trans. Med. Imaging.

[30] Apostolopoulos, S., Ciller, C., De Zanet, S. I., Wolf, S. & Sznitman, R. RetiNet: Automatic AMD identification in OCT volumetric data. *ArXiv* 1610.03628 (2016).

[31] Joskowicz, L., Cohen, D., Caplan, N. & Sosna, J. Inter-observer variability of manual contour delineation of structures in CT. *Eur*. *Radiol*. (2018). 10.1007/s00330-018-5695-5 30194472

[32] Domalpally A, Trane R, Reimers J, Blodi BA (2018). Evaluation of diabetic retinopathy using the ETDRS severity scale – is there a gold standard?. Invest. Ophthalmol. Vis. Sci..

[33] DeCroos FC (2012). Optical coherence tomography grading reproducibility during the comparison of age-related macular degeneration treatments trials. Ophthalmology.

[34] Menze BH (2015). The multimodal brain tumor image segmentation benchmark (BRATS). IEEE Trans. Med. Imaging.

[35] Schwarzer, P., Ebneter, A., Munk, M., Wolf, S. & Zinkernagel, M. S. One-Year results of using a Treat-and-Extend regimen without a loading phase with Anti-VEGF agents in patients with Treatment-Naive diabetic macular edema. *Ophthalmologica* 1–6 (2019). 10.1159/000495623 30654365

[36] Arendt P (2019). Exist strategy in a treat-annd-extend region for exudative age-related macular degeneration. Retina.

[37] Munk MR (2018). The impact of the vitreomacular interface in neovascular Age-Related macular degeneration in a Treat-and-Extend regimen with exit strategy. Ophthalmology Retina.

