# Supplementary material for: Expert-level Automated Biomarker Identification in Optical Coherence Tomography Scans
Source: Sci Rep. 2019 Sep 19;9:13605. doi: 10.1038/s41598-019-49740-7 (PMC6753124; doi:10.1038/s41598-019-49740-7)
Supplement: Supplementary file 1 — Supplementary Materials [file 41598_2019_49740_MOESM1_ESM.pdf]

Supplementary materials:
Expert-level Automated Biomarker Identification in Optical Coherence Tomography Scans

Thomas Kurmann[1,*], Siqing Yu[2], Pablo Marquez Neila[1], Andreas Ebneter[2], Martin Zinkernagel[2], Marion R. Munk[2], Sebastian Wolf[2], and Raphael Sznitman[1]

[1]ARTORG Center, University of Bern, Switzerland
[2]Department of Ophthalmology, Inselspital, University Hospital, University of Bern, Switzerland

Biomarker	Specification
Healthy	No pathology visible in the evaluated scan
Subretinal Fluid (SRF)	Well-defined darkening with a minimal horizontal extension of 100 um between the retinal pigment epithelium layer (RPE) and photoreceptor layer
Intraretinal Fluid (IRF)	Diffuse darkening and thickening of the neurosensory retina
Intraretinal Cysts (IRC)	Oval well-defined hyporeflective areas with a minimal extension of $25\mu m$ in any direction between the internal limited membrane and the photoreceptor layer
Hyperreflective Foci (HF)	Small points of increase reflectivity scattered throughout all retinal layers, primarily found in near vicinity of intraretinal cystoid spaces. They size of HF can vary from $25\mu m$ in diameter to $50\mu m$ and they can be clustered
Drusen	Located between the RPE and Bruch's membrane. They can appear hyper-reflective with some areas of hypo-reflectivity within the lesion depending on the composition. Bruch's membrane can be visible because the drusen is located between the RPE and Bruch's membrane. Drusen on the RPE can appear irregular, thinner and disrupted
Reticular Pseudodrusen (RPD)	Subretinal drusenoid deposits are seen in the subretinal space between the photoreceptors and the RPE. A typical sign of reticular pseudodrusen is an undulation of the ellipsoid zone. They are subretinal deposits, located above RPE in contrast to traditional drusen, which are located below the RPE
Epiretinal Membrane (ERM)	Thickening of the surface of the retinal nerve fiber layer (RNFL) within the whole macular cube scan. A hyperreflective line between the vitreomacular interface and the RNFL is visible
Geographic Atrophy (GA)	SD-OCT scans of geographic atrophy reveals RPE thinning, loss of ellipsoid zone (EZ) and interdigitation zone (IZ) lines, subsiding of the inner retinal layers as the outer layers are lost, and increased reflectivity of the Bruch's membrane and the choroid. The inclusive criteria are: (1) region of hypertransmission of at least 250 um in diameter in any lateral dimension, (2) zone of attenuation or disruption of the RPE of at least 250 um in diameter, and (3) evidence of overlying photoreceptor degeneration 9. Features of photoreceptor degeneration include all of the following: loss of the interdigitation zone, ellipsoid zone, and external limiting membrane and thinning of the outer nuclear layer
Outer Retinal Atrophy (ORA)	Defined by continuous non-visibility of the EZ and interdigitation zone (IZ) and severe thinning of the outer retina, in the setting of an intact RPE band. Hypertransmission associated with RPE degeneration is intermittent
Fibrovascular PED (FPED)	Detachment of the RPE from Bruch's membrane characterized by a hyperreflective structure underneath the RPE. Often the fibrovascular PED presents as undulating RPE and a "low-lying" PED, referred to as "double layer sign"

Table 1. Description and definitions of biomarkers annotated

Block	Layer	Input	Parameters
Conv	BatchNorm	*	decay, epsilon
	ReLu	*	-
	$Conv2d_1$	*	$kernel_1$, $channels_1$, $stride_1$, $dilation_1$
	BatchNorm	*	decay, epsilon
	ReLu	*	-
	$Conv2d_2$	*	$kernel_2$, $channels_2$, $stride_2$, $dilation_2$
	Add	Input, $Conv2d_2$	-
ConvSkip	BatchNorm	*	decay, epsilon
	ReLu	*	-
	$Conv2d_1$	*	$kernel_1$, $channels_1$, $stride_1$, $dilation_1$
	BatchNorm	*	decay, epsilon
	ReLu	*	-
	$Conv2d_2$	*	$kernel_2$, $channels_2$, $stride_2$, $dilation_2$
	$Conv2d_{skip}$	Input	$kernel_{skip}$, $channels_{skip}$, $stride_{skip}$, $dilation_{skip}$
	Add	$Conv2d_{skip}$, $Conv2d_2$	-

Table 2. Convolutional block definitions, (*) indicates the previous layer

Block	Name	Layer	Input	Parameters
0	block_0/conv2d	Conv2d	Input image	Kernel: 7x7, Channels: 16, Stride: 1, Dilation: 1
1	block_1/1	ConvSkip	*	Kernel$_1$:3x3, Channels$_1$:32, Stride$_1$:1, Dilation$_1$: 1<br>Kernel$_2$:3x3, Channels$_2$:32, Stride$_2$:1, Dilation$_2$: 1<br>Kernel$_{skip}$:1x1, Channels$_{skip}$:32, Stride$_{skip}$:1,Dilation$_{skip}$: 1<br>decay: 0.9, epsilon=2e-5
2	block_2/1	ConvSkip	*	Kernel$_1$:3x3, Channels$_1$:64, Stride$_1$:2, Dilation$_1$: 1<br>Kernel$_2$:3x3, Channels$_2$:64, Stride$_2$:1, Dilation$_2$: 1<br>Kernel$_{skip}$:1x1, Channels$_{skip}$:64, Stride$_{skip}$:2,Dilation$_{skip}$: 1<br>decay: 0.9, epsilon=2e-5
3	block_3/1	ConvSkip	*	Kernel$_1$:3x3, Channels$_1$:128, Stride$_1$:2, Dilation$_1$: 1<br>Kernel$_2$:3x3, Channels$_2$:128, Stride$_2$:1, Dilation$_2$: 1<br>Kernel$_{skip}$:1x1, Channels$_{skip}$:128, Stride$_{skip}$:2, Dilation$_{skip}$: 1<br>decay: 0.9, epsilon=2e-5
	block_3/2	Conv	*	Kernel$_1$:3x3, Channels$_1$:128, Stride$_1$:1, Dilation$_1$: 1<br>Kernel$_2$:3x3, Channels$_2$:128, Stride$_2$:1, Dilation$_2$: 1<br>decay: 0.9, epsilon=2e-5
	block_3/3	Conv	*	Kernel$_1$:3x3, Channels$_1$:128, Stride$_1$:1, Dilation$_1$: 1<br>Kernel$_2$:3x3, Channels$_2$:128, Stride$_2$:1, Dilation$_2$: 1<br>decay: 0.9, epsilon=2e-5
4	block_4/1	ConvSkip	*	Kernel$_1$:3x3, Channels$_1$:256, Stride$_1$:2, Dilation$_1$: 1<br>Kernel$_2$:3x3, Channels$_2$:256, Stride$_2$:1, Dilation$_2$: 1<br>Kernel$_{skip}$:1x1, Channels$_{skip}$:256, Stride$_{skip}$:2,Dilation$_{skip}$: 1<br>decay: 0.9, epsilon=2e-5
	block_4/2	Conv	*	Kernel$_1$:3x3, Channels$_1$:256, Stride$_1$:1, Dilation$_1$: 1<br>Kernel$_2$:3x3, Channels$_2$:256, Stride$_2$:1, Dilation$_2$: 1<br>decay: 0.9, epsilon=2e-5
	block_4/3	Conv	*	Kernel$_1$:3x3, Channels$_1$:256, Stride$_1$:1, Dilation$_1$: 1<br>Kernel$_2$:3x3, Channels$_2$:256, Stride$_2$:1, Dilation$_2$: 1<br>decay: 0.9, epsilon=2e-5
	block_4/4	Conv	*	Kernel$_1$:3x3, Channels$_1$:256, Stride$_1$:1, Dilation$_1$: 1<br>Kernel$_2$:3x3, Channels$_2$:256, Stride$_2$:1, Dilation$_2$: 1<br>decay: 0.9, epsilon=2e-5
5	block_5/1	ConvSkip	*	Kernel$_1$:3x3, Channels$_1$:256, Stride$_1$:1, Dilation$_1$: 2<br>Kernel$_2$:3x3, Channels$_2$:256, Stride$_2$:1, Dilation$_2$: 2<br>Kernel$_{skip}$:1x1, Channels$_{skip}$:256, Stride$_{skip}$:1,Dilation$_{skip}$: 1<br>decay: 0.9, epsilon=2e-5
	block_5/2	Conv	*	Kernel$_1$:3x3, Channels$_1$:256, Stride$_1$:1, Dilation$_1$: 2<br>Kernel$_2$:3x3, Channels$_2$:256, Stride$_2$:1, Dilation$_2$: 2<br>decay: 0.9, epsilon=2e-5
	block_5/3	Conv	*	Kernel$_1$:3x3, Channels$_1$:256, Stride$_1$:1, Dilation$_1$: 2<br>Kernel$_2$:3x3, Channels$_2$:256, Stride$_2$:1, Dilation$_2$: 2<br>decay: 0.9, epsilon=2e-5
	block_5/4	Conv	*	Kernel$_1$:3x3, Channels$_1$:512, Stride$_1$:1, Dilation$_1$: 2<br>Kernel$_2$:3x3, Channels$_2$:512, Stride$_2$:1, Dilation$_2$: 2<br>decay: 0.9, epsilon=2e-5
	block_5/5	Conv	*	Kernel$_1$:3x3, Channels$_1$:512, Stride$_1$:1, Dilation$_1$: 2<br>Kernel$_2$:3x3, Channels$_2$:512, Stride$_2$:1, Dilation$_2$: 2<br>decay: 0.9, epsilon=2e-5
	block_5/6	Conv	*	Kernel$_1$:3x3, Channels$_1$:512, Stride$_1$:1, Dilation$_1$: 2<br>Kernel$_2$:3x3, Channels$_2$:512, Stride$_2$:1, Dilation$_2$: 2<br>decay: 0.9, epsilon=2e-5
6	block_6/1	Conv	*	Kernel$_1$:3x3, Channels$_1$:512, Stride$_1$:1, Dilation$_1$: 4<br>Kernel$_2$:3x3, Channels$_2$:512, Stride$_2$:1, Dilation$_2$: 4<br>decay: 0.9, epsilon=2e-5
	block_6/2	Conv	*	Kernel$_1$:3x3, Channels$_1$:512, Stride$_1$:1, Dilation$_1$: 4<br>Kernel$_2$:3x3, Channels$_2$:512, Stride$_2$:1, Dilation$_2$: 4<br>decay: 0.9, epsilon=2e-5
	block_6/3	Conv	*	Kernel$_1$:3x3, Channels$_1$:512, Stride$_1$:1, Dilation$_1$: 4<br>Kernel$_2$:3x3, Channels$_2$:512, Stride$_2$:1, Dilation$_2$: 4<br>decay: 0.9, epsilon=2e-5
	block_6/4	Conv	*	Kernel$_1$:3x3, Channels$_1$:512, Stride$_1$:1, Dilation$_1$: 4<br>Kernel$_2$:3x3, Channels$_2$:512, Stride$_2$:1, Dilation$_2$: 4<br>decay: 0.9, epsilon=2e-5
7	block_7/1	Conv	*	Kernel$_1$:3x3, Channels$_1$:512, Stride$_1$:1, Dilation$_1$: 2<br>Kernel$_2$:3x3, Channels$_2$:512, Stride$_2$:1, Dilation$_2$: 2<br>decay: 0.9, epsilon=2e-5
8	block_8/1	Conv	*	Kernel$_1$:3x3, Channels$_1$:512, Stride$_1$:1, Dilation$_1$: 1<br>Kernel$_2$:3x3, Channels$_2$:512, Stride$_2$:1, Dilation$_2$: 1<br>decay: 0.9, epsilon=2e-5
Pooling	pooling/bnorm	BatchNorm	*	decay: 0.9, epsilon=2e-5
	pooling/relu	ReLu	*	-
	pooling/max_pooling	Max Pooling	*	-
	pooling/avg_pooling	Avg Pooling	pooling/relu	-
	pooling/concat	Concatenate	pooling/max_pooling<br>pooling/avg_pooling	-
FC	fc/fc	Fully Connected	*	Outputs: 11

Table 3. Proposed network specification and its block configuration. (*) indicates the previous layer

	**Healthy**	**SRF**	**IRF**	**HF**	**Drusen**	**RPD**	**ERM**	**GA**	**ORA**	**IRC**	**FPED**
Training Set (23’030)	6480	1142	2947	5668	5077	1995	6139	1093	2280	4321	4766
Test Set (1029)	165	65	48	178	376	153	140	62	200	54	359

Table 4. Distribution of biomarkers in majority voted validation set

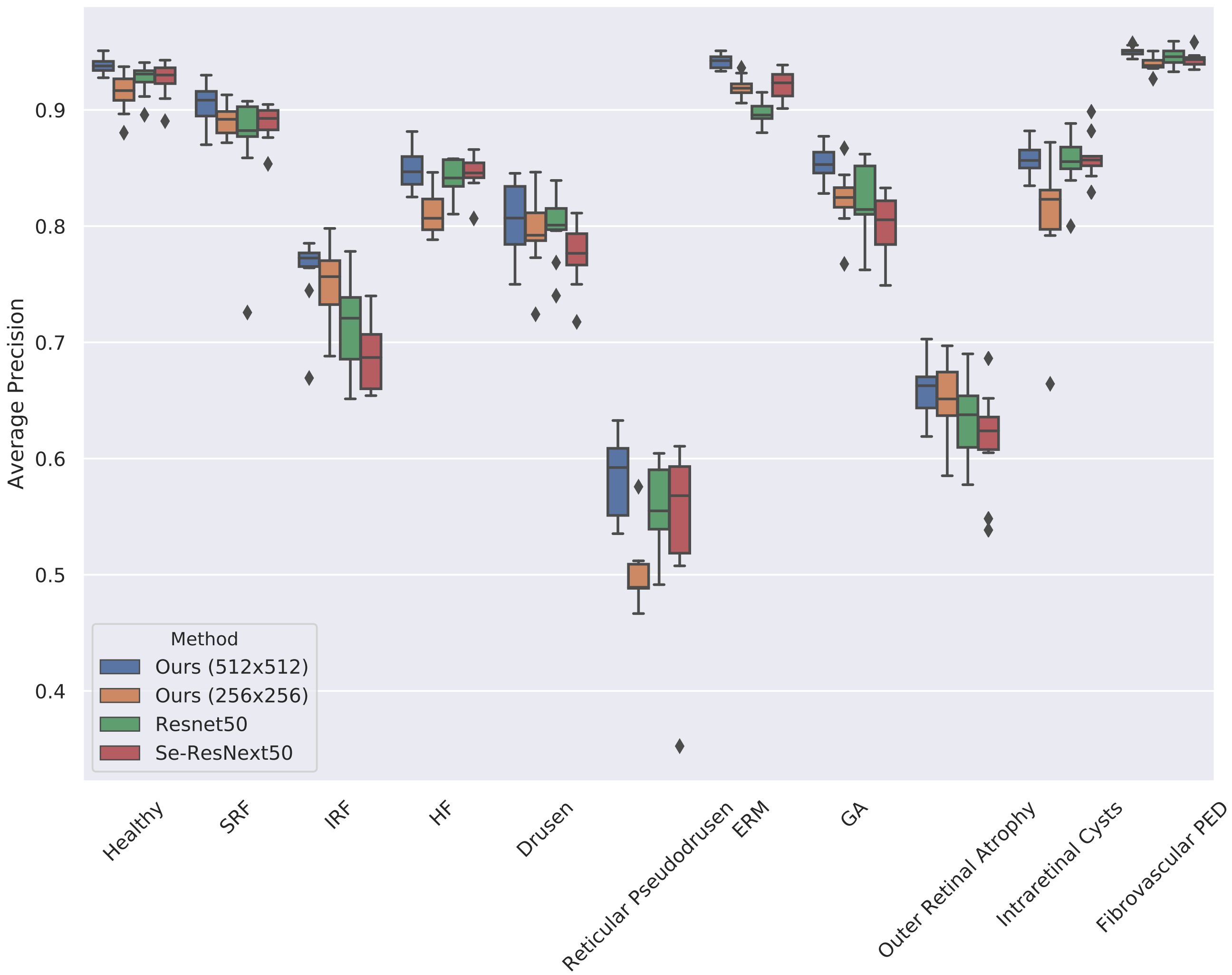

Figure 1. 10 fold cross validation results of comparison of network architectures on biomarker Bscan classification. Different standard architectures are shown: ResNet-50[1],Ours, SE-ResNext[2]. In addition, we evaluate the impact of using two image size resolutions: 256×256px and 512×512px. All networks are pre-trained on Imagenet. The mean average precision of ResNet-50 is 0.825 ± 0.013, Ours 0.840 ± 0.01 and Se-ResNext50 0.816 ± 0.0084. Our proposed network outperform other networks with similar performance on the ImageNet classification task. The mean biomarker average precision over 10 folds is $0.813 + / - 0.015$ for 256×256 and 0.840 ± 0.010 for 512×512. No biomarkers performed better with the lower resolution. The largest difference in average precision is found for Reticular Pseudodrusen (+14.3%), Intraretinal Cysts (+5.94%) and HF (+4.39%). All three biomarkers are small to medium size in appearance, hence showing that by increasing the input resolution we are able to capture more small scale features whilst retaining a large receptive field required to capture global context.

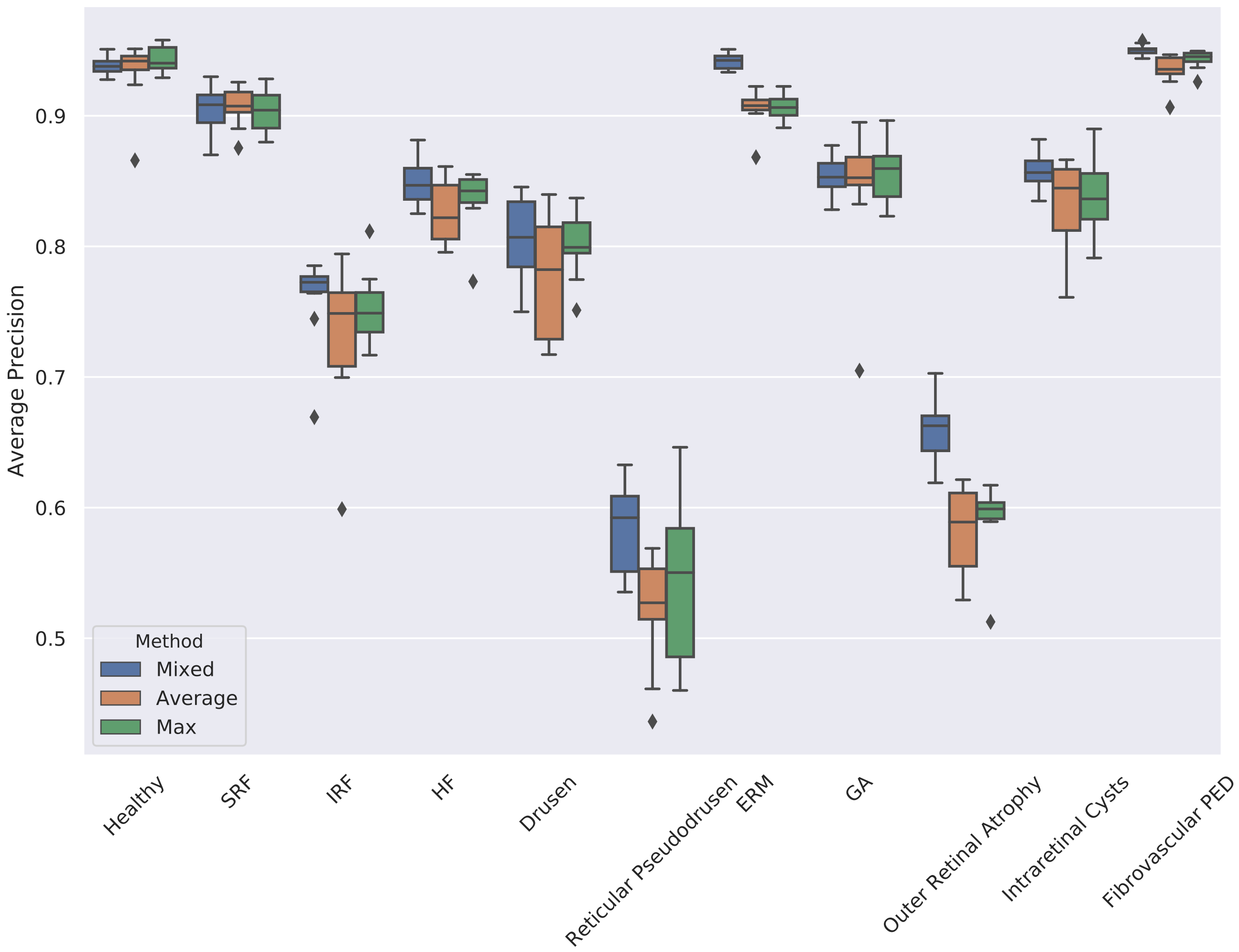

Figure 2. Comparison of global pooling strategies. We train three 10 fold cross validated models using average, max and mixed pooling. The average precision of all biomarkers for average pooling is 0.810 ± 0.026, for max pooling 0.824 ± 0.10 and 0.840 ± 0.10 for mixed pooling. The performance of small biomarkers such as HF, Drusen, ERM and Reticular Pseudodrusen are lower in the average pooling case, confirming our intuition that average pooling is ill suited for our problem at hand. Interestingly max pooling performs better for the healthy biomarker than mixed pooling. One reason for this increased performance can be explained by looking at the inverse problem, if a single pixel is not healthy in a scan, the biomarker healthy can not be considered, thus making it easier for the network to learn with max pooling.

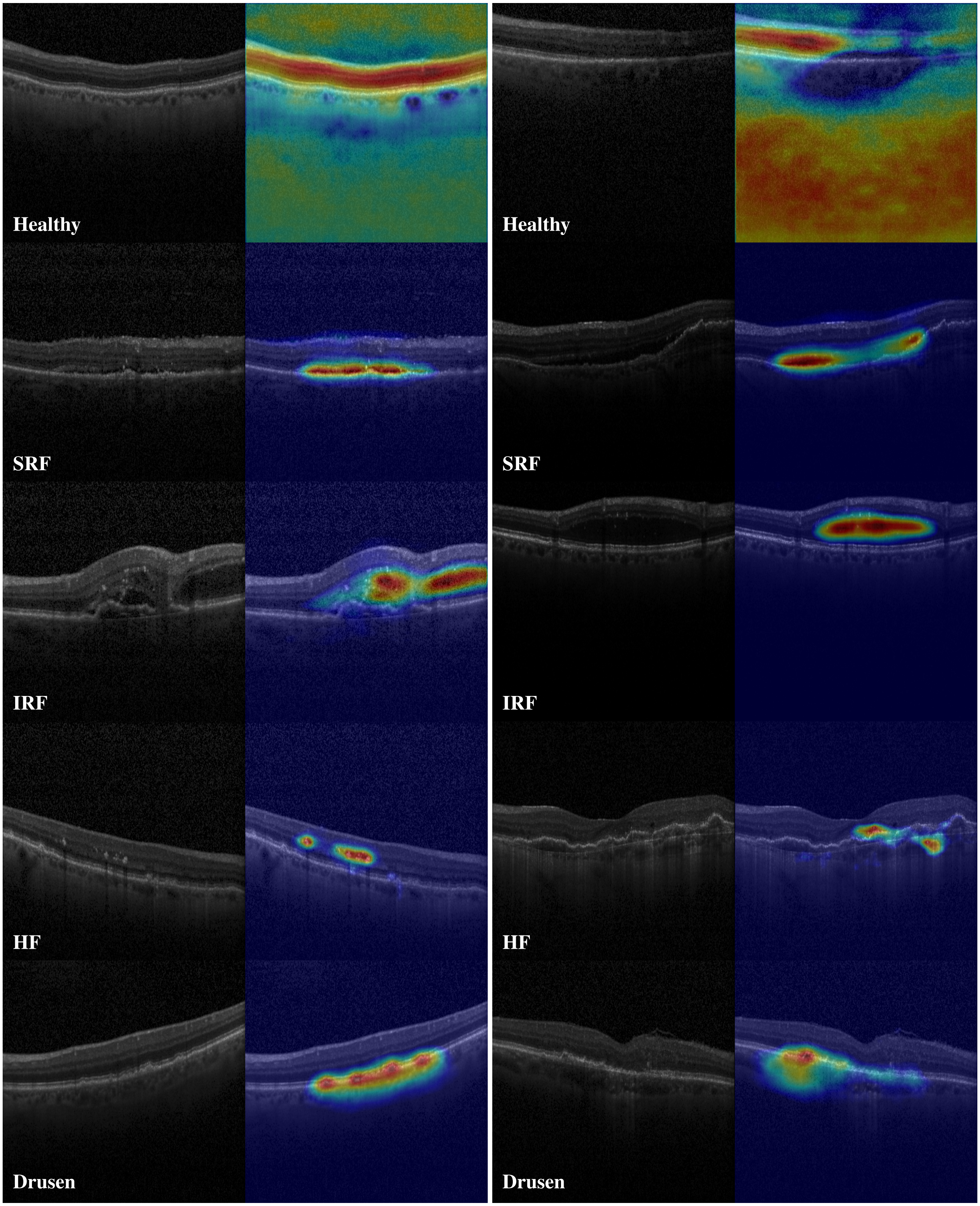

Figure 3. Classifier activation maps for each biomarker: Good examples (**left**), Poor examples (**right**). As our network retains a spatial size of 64x64 pixels in the last layer, we increase the quality of the activation maps by applying test time augmentation and average the resulting activations and gradients. Red regions illustrates regions of the image that are automatically selected by the method to correctly predict the corresponding biomarker.

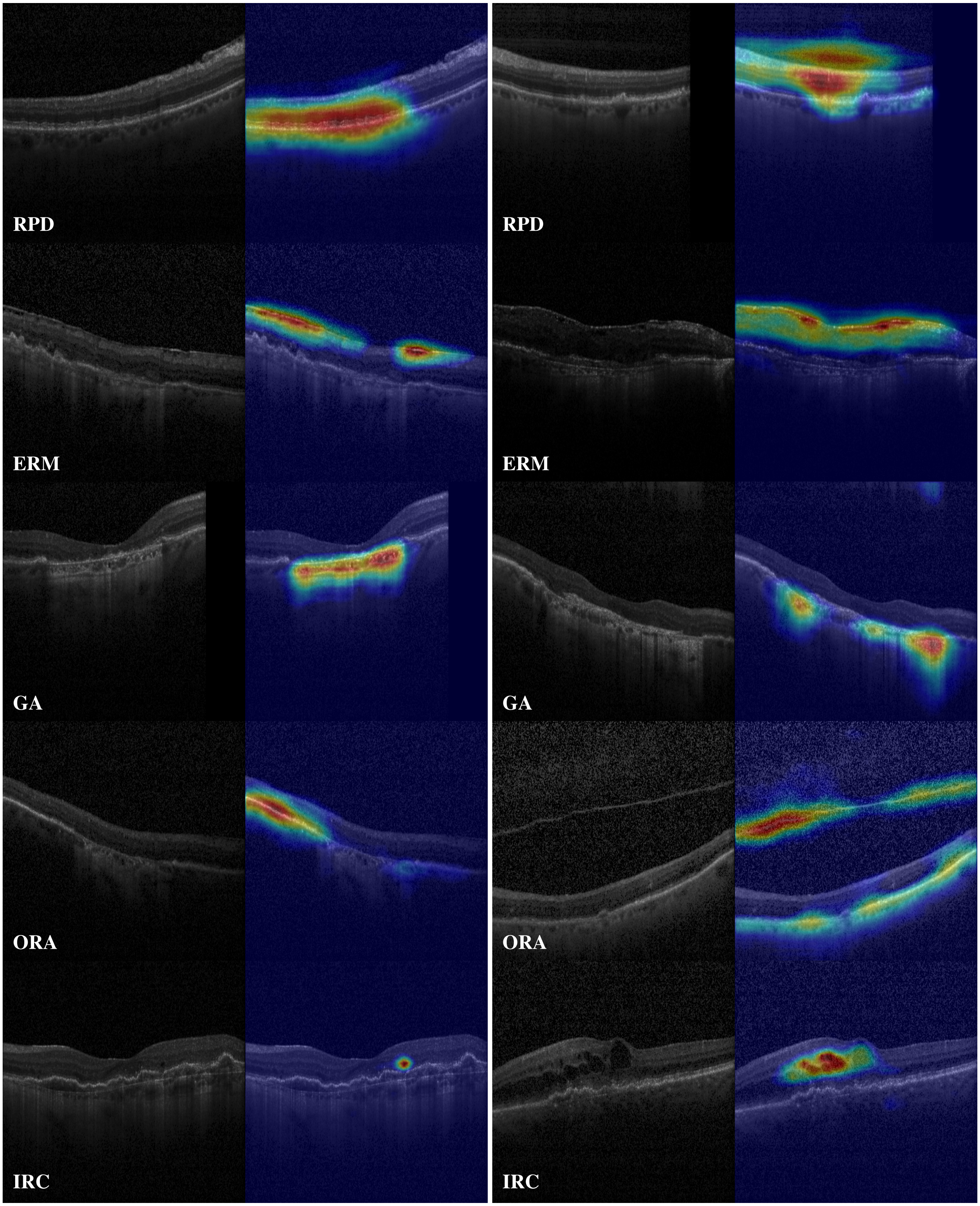

Figure 4. Classifier activation maps for each biomarker: Good examples (**left**), Poor examples (**right**). As our network retains a spatial size of 64x64 pixels in the last layer, we increase the quality of the activation maps by applying test time augmentation and average the resulting activations and gradients. Red regions illustrates regions of the image that are automatically selected by the method to correctly predict the corresponding biomarker.

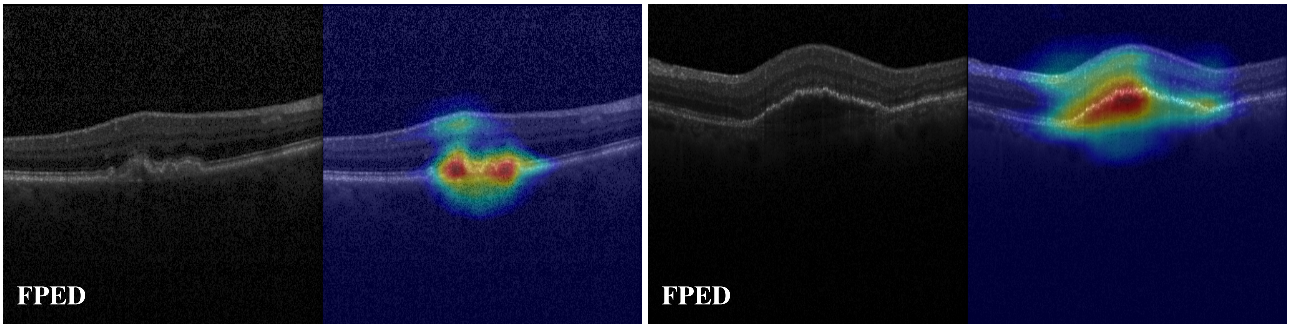

Figure 5. Classifier activation maps for each biomarker: Good examples (**left**), Poor examples (**right**). As our network retains a spatial size of 64x64 pixels in the last layer, we increase the quality of the activation maps by applying test time augmentation and average the resulting activations and gradients. Red regions illustrates regions of the image that are automatically selected by the method to correctly predict the corresponding biomarker.

References

1. He, K., Zhang, X., Ren, S. & Sun, J. Deep residual learning for image recognition. *ArXiv* (2015). 1512.03385.

2. Hu, J., Shen, L., Albanie, S., Sun, G. & Wu, E. Squeeze-and-Excitation networks. *ArXiv* (2017). 1709.01507.
